# Intracellular and Plasma Membrane Events in Cholesterol Transport and Homeostasis

**DOI:** 10.1155/2018/3965054

**Published:** 2018-08-06

**Authors:** Dmitry Y. Litvinov, Eugeny V. Savushkin, Alexander D. Dergunov

**Affiliations:** National Research Centre for Preventive Medicine, 10 Petroverigsky Street, 101990 Moscow, Russia

## Abstract

Cholesterol transport between intracellular compartments proceeds by both energy- and non-energy-dependent processes. Energy-dependent vesicular traffic partly contributes to cholesterol flux between endoplasmic reticulum, plasma membrane, and endocytic vesicles. Membrane contact sites and lipid transfer proteins are involved in nonvesicular lipid traffic. Only “active" cholesterol molecules outside of cholesterol-rich regions and partially exposed in water phase are able to fast transfer. The dissociation of partially exposed cholesterol molecules in water determines the rate of passive aqueous diffusion of cholesterol out of plasma membrane. ATP hydrolysis with concomitant conformational transition is required to cholesterol efflux by ABCA1 and ABCG1 transporters. Besides, scavenger receptor SR-B1 is involved also in cholesterol efflux by facilitated diffusion via hydrophobic tunnel within the molecule. Direct interaction of ABCA1 with apolipoprotein A-I (apoA-I) or apoA-I binding to high capacity binding sites in plasma membrane is important in cholesterol escape to free apoA-I. ABCG1-mediated efflux to fully lipidated apoA-I within high density lipoprotein particle proceeds more likely through the increase of “active” cholesterol level. Putative cholesterol-binding linear motifs within the structure of all three proteins ABCA1, ABCG1, and SR-B1 are suggested to contribute to the binding and transfer of cholesterol molecules from cytoplasmic to outer leaflets of lipid bilayer. Together, plasma membrane events and intracellular cholesterol metabolism and traffic determine the capacity of the cell for cholesterol efflux.

## 1. Introduction

Cholesterol homeostasis is a well-coordinated machinery of* de novo *cholesterol synthesis in endoplasmic reticulum and uptake of cholesterol-containing low-density lipoproteins (LDL). Cholesterol synthesis is under tight control by lipoprotein-derived cholesterol that includes inhibition of the sterol regulatory element-binding protein (SREBP) pathway with the decreased expression of genes involved in cholesterol synthesis and uptake [1]. Cholesterol turnover is normally balanced by cholesteryl ester formation at cholesterol excess and cellular cholesterol efflux by both passive and active transport. Reverse cholesterol transport from the cell to the liver is considered as a major atheroprotective event with cholesterol efflux as a rate-limiting step [2, 3]. Eukaryotic cells maintain a gradient in sterol concentration between plasma membrane (PM) and the membranes of cell organelles such as endoplasmic reticulum (ER) by both vesicular and nonvesicular mechanisms involving lipid transport proteins. PM contains 40-90% of total cellular cholesterol [4], for example, 64% in CHO cells [5] and 90% in human fibroblasts and FU5AH rat hepatoma cells [6, 7]. ER is the site of cholesterol synthesis; however in contrast to PM, the ER contains just a small fraction of total cell cholesterol, such as 0.5% of total cell cholesterol in human fibroblasts [8]. Such a drastic difference in cholesterol pools in the PM and ER is due to much higher cholesterol concentration in PM compared to ER and other intracellular membranes. The fraction of cholesterol in PM relative to total PM lipids is 30-40 mol% in leukocytes, epithelial cells, neurons, and mesenchymal cell [9]. The sterol/phospholipid ratio in RAW264.7 macrophages and mouse fibroblast LM cells is 0.5-0.6 for PM, as compared to 0.25-0.3 for ER, and 0.05-0.1 for mitochondrial membrane [10, 11]. The ratio of cholesterol to combined cholesterol and phospholipid (PL) content for ER for CHO cells is 5-7 mol%, compared to 35 mol% for the whole cell [12, 13]. All other intracellular membranes have smaller cholesterol concentration than the PM. Importantly, the PM cholesterol is the cholesterol that participates in the efflux to the extracellular acceptors [14]. Despite the large size of PM cholesterol pool, efflux of just a small fraction of PM cholesterol can be significantly affected by intracellular events. Indeed, while cholesterol efflux in cholesterol-depleted RAW 264.7 cells is as small as less than 1% of total cholesterol, the knockdown of dynamin-2, or ADP-ribosylation factor 6 (Arf6), or Cdc42 reduces it even further [15]. The efflux of 2% of total cell cholesterol in THP-1 cells was almost completely inhibited by the knockdown of oxysterol-binding protein-related protein 6 (OSBPL6, aka ORP6) [1]. The efflux of 4% of total cholesterol in peritoneal macrophages from WT mouse was reduced by 2-fold in the peritoneal macrophages with knockout in Niemann-Pick disease, type C1 (NPC1) protein [16]. Another illustration of the effect of intracellular or PM processes on cholesterol efflux is the overexpression of caveolin-1 in RAW 264.7 that increases cholesterol efflux to HDL from 2.5% to almost 5% [17]. The lipid composition and structural features of plasma membrane [18, 19] also contribute to cholesterol efflux.

The goal of this review is to describe the complex processes of cholesterol metabolism and cholesterol traffic inside the cell and the effect of these processes on the cholesterol efflux from the cells. The mechanisms of cholesterol transfer between cell membranes and underlying reason of gradient of cholesterol concentration between intracellular and plasma membranes will be discussed. We also describe four known mechanisms of cholesterol efflux–aqueous diffusion, facilitated diffusion mediated by SR-B1 receptor, and active unidirectional efflux mediated by ABCA1 and ABCG1 transporters. The contribution of different pools of cholesterol and types of acceptor will be also considered.

## 2. Lipid Rafts and Cholesterol Pools in Lipid Bilayer and Cell Membranes

### 2.1. Membrane Lipid Composition and Two Kinetic Pools of PM Cholesterol

**Figure 1:**
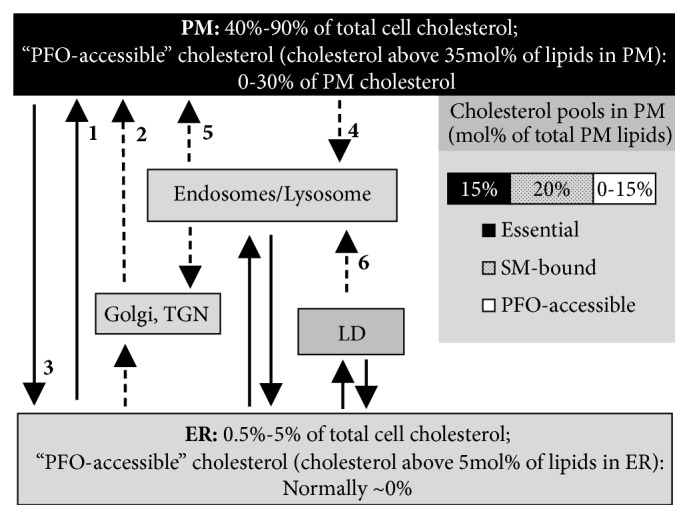
Cholesterol distribution and movement between major compartments. Solid arrows indicate nonvesicular cholesterol transport (including transport via membrane contact sites and lipid transfer proteins); dashed arrows indicate vesicular transport and transport of cholesterol mediated by organelles. The numbers indicate the following: (1) the major nonvesicular, not cytoskeleton-dependent, energy-dependent path of cholesterol transport from ER to PM; (2) the minor vesicular transport from ER to PM via Golgi; (3) not energy-dependent, cytoskeleton-dependent cholesterol transport from PM to ER; (4) transport of LDL bound by LDL receptor in clathrin-coated vesicles; (5) energy-dependent, cytoskeleton-dependent cholesterol transport via lysosome-related organelles; (6) lysosomal degradation of LD. Three pools of cholesterols with different accessibility to water phase are known in PM. PFO-accessible cholesterol is the most available pool for the interactions with reagents in aqueous phase, such as PFO (Perfringolysin O), cholesterol oxidase, and cyclodextrin. This pool with variable size is considered as a putative “active” cholesterol. The size of this pool is very small at cholesterol depletion, while at the increase of its size in PM cholesterol moves to the ER. PFO-accessible pool in ER appears at much smaller mol% of cholesterol. PM (plasma membrane), ER (endoplasmic reticulum), LD (lipid droplets), TGN (trans-Golgi network), LDL (low-density lipoprotein), and SM (sphingomyelin).

The distribution of lipids between membrane leaflets is not even. The cytosolic leaflet of PM is enriched with PE, PI, and PS compared to outward leaflet and is poorer in sphingolipids and PC [10, 20]. In addition, the composition of the membranes is not isotropic because of rafts and other structural features [21]. Dependent on the membrane composition, cholesterol can be associated with other lipids in more or less tight and stable complexes that affect its activity. The ability to form a complex and its stoichiometry depends on the lipid structure, such as the saturation and length of the acyl chains and the size of the polar head. The lipid:cholesterol molar ratio in the complexes usually varies between 3:1 and 1:1. Cholesterol preferably interacts with lipids containing large polar heads and saturated fatty acid residues due to increased shielding of cholesterol from the aqueous phase by large heads and a stronger Van der Waals interaction with saturated chains [18]. Under certain conditions, the addition of cholesterol to phospholipids leads to phase separation: phosphatidylcholine and especially sphingomyelin concentrate in the ordered and condensed cholesterol-rich phase, while the remaining phospholipids are displaced into the liquid phase. The pressure-composition phase diagram for cholesterol-phospholipid mixtures is characterized by an initial increase in pressure at low cholesterol content caused by the formation of ordered regions in the liquid phase. As cholesterol content increases, the pressure decreases due to the formation of a stoichiometric complex. The pressure then increases again when the liquid regions are formed in the condensed phase with a further increase in the cholesterol content [19]. Cholesterol, not associated in complexes with other lipids, is more available for reactions and more active at the escape from the membrane, probably due to disruption of its orientation in the bilayer. Cholesterol molecules with higher chemical activity are more accessible for the removal by cholesterol acceptors, for oxidation by cholesterol oxidase, and for binding to bacterial protein toxins of cholesterol-dependent cytolysins family, e.g., Perfringolysin O (PFO) and anthrolysin [19, 22, 23]. In the membrane, cholesterol pools with different activity can simultaneously be present (Figure 1). It was suggested that cholesterol distribution in membranes of various organelles depends on the composition of these membranes and corresponds to the stoichiometry of the cholesterol complexes. Two existing views on uneven cholesterol content in plasma and intracellular membranes suggest (1) the distribution of cholesterol by diffusion or assisted diffusion of cholesterol, which depends on cholesterol concentration and lipid composition of the membranes, and (2) the participation of energy-dependent active transport of cholesterol by cholesterol transfer proteins [19, 24]. Cholesterol level in endoplasmic reticulum does not exceed 5% and there is no complex with 19:1 stoichiometry; the complex formation is discarded for such phospholipid as 1-stearoyl-2-oleoyl-sn-glycero-3-phosphatidylcholine. Alternatively, the diminished cholesterol content may originate from the presence in ER of any substances that exclude cholesterol from the complex.

Cholesterol level is tightly maintained in cell membrane due to complex formation. Thus, the excess of cholesterol in the PM results in the cholesterol efflux or its transfer to ER for conversion to CE or in some cases to mitochondria for oxidation to oxysterols [25]. Normally, the cholesterol content of PM of fibroblasts and CHO cells is about 40-50% of total PM lipids. The association of plasma membrane cholesterol with a mutant form of bacterial Perfringolysin O (PFO*∗*) occurs at cholesterol increase over 35%. Thus up to ~15% are available for the binding to the PFO*∗*. This cholesterol pool disappeared when cells are cholesterol-depleted. The treatment of the cells with sphingomyelinase releases cholesterol from cholesterol-SM complexes thus adding 10-23% of the cholesterol to the PFO*∗*-accessible pool. The rest of the cholesterol does not bind to PFO*∗*. It is an essential pool, and the depletion of this cholesterol results in the rounding of the cells and their detachment to the medium. The binding of ER cholesterol with PFO occurs at cholesterol level over 5%. The relation between static and kinetic cholesterol pools remains undetermined; however, the concentration of cholesterol complex with sphingomyelin decreased at the increase of cyclodextrin content [14, 26].

Two pools of cholesterol are observed in the kinetics of cholesterol efflux to cyclodextrin in Fu5AH hepatoma cells, mouse fibroblasts L-cells, human skin fibroblasts, and CHO-K1 cells. The fast pool is 20-60% (the highest is for the Fu5AH cells) of cell cholesterol and the efflux half time is about 15-23 sec. The slow pool is 50-80% and its half time of efflux is about 15-35 min [14, 27]. The cholesterol efflux to HDL_3_ in CHO-K1 cells similarly to the efflux to cyclodextrin shows fast and slow pools. However, the sizes of both pools for the efflux to HDL3 are much smaller than if the cholesterol acceptor is cyclodextrin. The temperature dependence of cholesterol efflux indicates that activation energy for cholesterol transfer to HDL_3_-derived apoHDL-PC is 20 kcal/mol, which is much higher than for cyclodextrin (7 kcal/mol). Slow and fast pools exchange cholesterol with a half time 20-30 min. All cholesterol molecules of the fast pool and the majority of cholesterol of the slow pool escape from the PM [14, 27].

Energy poisons do not significantly affect the fast pool of cholesterol. Sodium azide (NaN_3_), potassium cyanide (KCN), sodium fluoride (NaF), ATPase inhibitor bafilomycin A1, or mixture of NaN_3_ with 2-deoxyglucose does not affect half-times and size of fast cholesterol pool in CHO-K1 cells or just slightly affects them in CHO cells expressing human transferrin receptor [14, 28]. The data on the effect of energy poisons on the parameters of slow cholesterol pool are controversial. NaN_3_, KCN, NaF, and ATPase inhibitor bafilomycin A1 do not affect half time and size of slow cholesterol pool in CHO-K1 cells [14]. However, energy poison mixture of NaN_3_ with 2-deoxyglucose effectively prevents cholesterol efflux from the slow pool in CHO cells expressing human transferrin receptor [28].

### 2.2. Cholesterol Homeostasis Might Be Regulated by “Active” Cholesterol

The difference in the chemical activity of cholesterol between various membranes results in the cholesterol flux from one membrane to another [22]. Sphingomyelins (SMs) are known as lipids that can be associated with cholesterol in membranes. Partial hydrolysis of SMs by treatment of the cells with sphingomyelinase results in rapid flux of cholesterol from PM to intracellular cholesterol pools [72]. It can be assumed that many processes that depend on individual cholesterol molecules, such as cholesterol diffusion and cholesterol-protein interaction, similarly depend on the concentration of the accessible “active” cholesterol, but not the total cholesterol. An increase of ER cholesterol above ~5 mol% results in the appearance of PFO-accessible cholesterol and in the disappearance of SREBP-2 in the nucleus controlled by SREBP cleavage-activating protein (SCAP). It suggests that SCAP protein senses the “active” cholesterol in the membrane and becomes activated in the presence of active cholesterol [12]. On the contrary, the “active” cholesterol inhibits the activity of ER-associated enzyme HMG-CoA reductase. Its activity is minimal at normal or increased “active” cholesterol in PM but increases when the cholesterol level is not enough for the appearance of the active cholesterol [73].

A model was proposed that explains sterol gradient between ER and PM and the ability of the sterol-poor ER to respond to the small changes in sterol content in the sterol-rich PM. It suggests that PM rafts contain the most of PM sterols, which is not “free” (or “active”) and does not participate in the exchange with ER cholesterol. “Free” (not raft-associated) cholesterol is transferred between ER and PM by nonvesicular mechanism. The concentration of “free” cholesterol in the ER and PM is about the same, and the difference in the total cholesterol content between ER and PM is caused by the removal of the active cholesterol into the lipid rafts [74]. The concentration of “active” cholesterol depends on the cholesterol concentration and the membrane composition [75]. Indeed, while PFO-sensitive pool of cholesterol in the ER appears at about 5 mol% of cholesterol, the PM needs around 35 mol% cholesterol before it starts to appear in PFO-sensitive pool [76]. Thus, the gradient of cholesterol concentration between the PM and ER might be thermodynamically stable.

## 3. Intracellular Cholesterol Turnover

A number of intracellular proteins affect cholesterol efflux to extracellular acceptors of cholesterol (Table 1) with activator and inhibitor properties toward cholesterol synthesis and uptake, cholesterol distribution in the membrane, intracellular vesicular and nonvesicular trafficking, cytoskeletal organization, and cholesterol escape. It can be assumed that proper cholesterol homeostasis and fully functional intracellular cholesterol transport are required for normal level of cholesterol efflux. The central processes that maintain cholesterol balance and are common for the most of the cells are described below.

### 3.1. Abundance of Cholesterol Pools

A person every day receives about 400 mg of cholesterol with food while secreting through the liver approximately 1 g [77]. The rate of cholesterol synthesis in humans is estimated at about 10 mg/day per kg of body weight. It is assumed that liver contributes roughly 10% of this rate and the rest of the synthesis occurs in intestine and peripheral tissues [78, 79]. Endogenous cholesterol as well as the majority of other lipids is synthesized by the ER. In particular, rate-limiting enzyme in cholesterol synthesis 3-hydroxy-3-methylglutaryl coenzyme A reductase (HMGCR) is located in the ER. Most cells also uptake cholesterol from lipoproteins in normal conditions [80]. Low-density lipoproteins are internalized by LDL receptor (LDLR) in clathrin-coated vesicles. The vesicle is transported to sorting endosome, where LDL dissociates from the LDL receptor and the latter is recycled back to the plasma membrane via the endocytic recycling compartment (ERC). LDL goes to late endosome (LE), where cholesteryl ester (CE) of LDL is hydrolyzed by acid lipase and resulting cholesterol is distributed mainly to PM, to ER to a lesser degree, and to cell membranes of other organelles [81, 82]. Niemann-Pick type C proteins NPC1 and NPC2 are critically important for unloading of cholesterol from LE. A small GTPase Rab8 participates in cholesterol transport from LE to PM in Myosin5-dependent movement of cholesterol-enriched lysosome-related organelles along actin cytoskeleton [82]. Depletion of Rab8 in foam cell inhibits cholesterol efflux to apoA-I in part by the reduction of ABCA1 level at the PM [46]. One of the routes of cholesterol transport from LE to ER is vesicular transport through trans-Golgi network (TGN) and requires NPC1 and v-SNARE vesicle-associated membrane protein 4. Peritoneal macrophages from NPC1 knockout mouse show less efficient cholesterol efflux compared to the normal peritoneal macrophages (Table 1). In addition, there are membrane contact sites (MCSs) between LE and ER with cholesterol-binding proteins ORP1L, ORP5, and STARD3 that can participate in cholesterol transfer [82]. While cholesterol content of ER is much lower than of PM, it plays essential role in maintaining cholesterol homeostasis as a site of sensing cholesterol level that regulates expression of* LDLR* and* HMGCR* and a site of cholesterol synthesis if required and esterification for storage when cholesterol is in excess. Loading of fibroblasts with cholesterol using hydroxypropyl-beta-cyclodextrin-cholesterol complex that increases total cell cholesterol by 50% results in 10-fold increase in cholesterol level in the ER from 0.5% to 5% of total cell cholesterol. A depletion of the cells by 25% decreased ER cholesterol by 75% of original level [8]. An excess of some intermediates of cholesterol synthesis results in HMGCR degradation. Enzyme structure has sterol-sensing domain (SSD) and it is subjected to proteasome degradation if it binds oxysterols, lanosterol, 4,25-dihydrolanosterol but not cholesterol [80]. On the contrary, acyl-coenzyme A:cholesterol acyltransferase (ACAT) is activated by cholesterol and at higher cholesterol level converts it to CE for storage [83].

Both the biosynthesis and uptake of cholesterol are transcriptionally regulated by sterol regulatory element-binding protein family of SREBP-1a, SREBP-1c, and SREBP-2. The function of SREBPs is dependent on proper protein trafficking from ER to Golgi. When cell does not require a supply of cholesterol, SREBPs are anchored to ER. At low cholesterol condition, sterol-sensing domain (SSD) of SREBP cleavage-activating protein (SCAP) loses cholesterol, and SCAP initiates COPII-mediated incorporation of SREBPs into budding vesicles and transport them from the ER to the Golgi, where SREBPs are cleaved by Site-1 and Site-2 proteases. N-termini of SREBPs release into cytoplasm and move to nucleus escorted by importin-beta, where they induce expression of* LDLR, HMGCR*, Insulin induced gene 1 (*Insig-1*), and other SREBP target genes. In addition, SREBPs suppress expression of* ABCA1* that removes cholesterol to extracellular acceptors. When cholesterol level becomes too high, SCAP/SREBP complex binds to ER-anchored Insig-1, which retains the complex in the ER and prevents induction of target genes [80].

When cells become depleted of cholesterol, they first start to utilize CE stored in lipid droplets (LD) instead of the synthesis of new cholesterol [80]. A large number of CE-rich LDs is an indicator of macrophage transformation to foam cell because of excessive uptake of cholesterol. Lipid droplets are organelles that store sterol esters, triglycerides, and some other neutral lipids. The neutral lipid core is surrounded by a monolayer of phospholipids that contains a number of proteins that participate in LD metabolism. LDs are formed in ER because of synthesis of neutral lipids, such as CE that is synthesized from newly synthesized or LDL-derived cholesterol and fatty acyl-CoA by ACAT-1 or sterol O-acyltransferase 1. As the concentration of neutral lipids increases, they cannot be dissolved anymore in the ER membrane, and supposedly, a “lens” of neutral lipids between ER membrane leaflets appears that grows to a “drop” being still attached to the ER membrane. An integral membrane protein Seipin is detected in the LD-ER contact site. It is not fully clear whether fully formed LD stays attached to the ER or separated from the ER. Nevertheless the proteins of COPI vesicle coats are found on the LD surface. COPI vesicle transports cargo from Golgi to ER [84–86]. In macrophage foam cells cholesterol is constantly esterified by ACAT and deesterified by neutral CE hydrolases, such as carboxylesterase 1 and, possibly, neutral cholesterol ester hydrolase 1 [87]. That “futile cycle” is a LD-based mechanism that can help to maintain the normal cholesterol concentration. Beside the cytoplasmic lipolysis, LD can release cholesterol by LD lysosomal degradation in an autophagy route when CE is hydrolyzed by lysosomal acid lipase. In foam cells, the LD incorporates into autophagosome that fuses with lysosome and releases cholesterol for efflux from the cells. Inhibition of autophagy by Atg5 gene knockout reduces cholesterol efflux to apoA-I and reverses cholesterol transport to the liver* in vivo*, and mTOR inhibitors that stimulate autophagy are atheroprotective [88, 89]. An inhibition or knockdown of ACAT-1 results in the increase of cholesterol:phospholipid ratio in PM rafts and in the stimulation of ABCA1-dependent cholesterol efflux [90]. Incubation of the cells with cholesterol acceptors depletes cellular CE content [91].

### 3.2. Vesicular and Nonvesicular Cholesterol Traffic

Intracellular vesicular movement includes lipid transfer from one compartment to another as a constituent of the membrane of secretory vesicles [92, 93]. Anterograde COPII (coat protein complex II) vesicles traverse from the ER to Golgi, and retrograde COPI vesicles traverse from Golgi to the ER. Golgi along with trans-Golgi network is structurally highly dynamic organelle and intra-Golgi vesicle transport is not clearly understood. The Golgi routes the vesicle to PM and endosomal compartments. Intracellular traffic between compartments depends on various mechanisms. These mechanisms can be roughly described as energy-dependent and cytoskeleton-dependent. For instance, vesicle transport requires both energy and functional cytoskeleton. Despite the hypothetical possibility of vesicular traffic to transport significant amount of cholesterol, this route plays just a minor role in the cholesterol transfer from the site of the synthesis (ER) to PM. The disruption of cytoskeleton has no effect on the cholesterol transfer from ER to PM, and disruption of vesicle traffic by brefeldin A decreases nascent cholesterol transport to PM just by 20% [94]. Newly synthesized cholesterol bypasses the esterification to CE and appears in PM with half time of about 15 min [7]. This transport is inhibited by energy poisons KCN + KF or NaN_3_ + NaF in both mammalian and yeast cells [74, 95]. The transport of nascent cholesterol to PM stops if the cells are cooled to 15°C. At this temperature, the nascent cholesterol accumulates in the ER, and after retuning cells to 37°C cholesterol transport to PM is restored [95]. The disruption of cytoskeleton by cytochalasin, colchicine, and nocodazole or the disruption of Golgi apparatus by monensin and brefeldin A just slightly inhibits cholesterol transport from ER to PM [4, 95]. Microtubule disruption reagent nocodazole inhibits the transport just by 25% and the disruption of Golgi by brefeldin A by 20% [96].

The PM cholesterol is constantly transported back to ER by not energy-dependent mechanism. This transport is inhibited by disruption of the cytoskeleton and acidic compartments [4]. It was estimated that if the whole PM pool of cholesterol participates in the cycling, then the half time of cholesterol cycling is 40 min [7]. Considering the hypothesis of some “active” cholesterol in membranes, the half time is even faster. Unlike creating a cholesterol gradient between PM and ER, its maintaining does not depend on energy. Incubation of the cells with energy poisons does not change the distribution of cholesterol between PM and ER [95, 97]. The rate of dehydroergosterol transport to cholesterol-enriched endocytic recycling compartment from the PM was not greatly affected by ATP depletion with energy poison mixture of NaN_3_ with 2-deoxyglucose, indicating that the transport was mainly nonvesicular [28]. Oppositely, the efflux of sterol from ERC to the PM is inhibited significantly by the energy poisons [28] indicating that vesicular transport out of, but not into, the ERC is a major contributor to sterol transport kinetics. However, recent data of the same group suggest rather different mechanism of bidirectional sterol movement. The poison mixture decreased the rate of PM to ERC and ERC to PM transfer by ~30%, so that ~70% of cholesterol flux is not dependent on vesicle transport in osteosarcoma U20S cells that stably express the scavenger receptor A SRA; the half time of dehydroergosterol transfer between PM and ERC is about 15 min in both directions [98]. In yeast, the disruption of Sec18p, the yeast orthologue of mammalian N-ethylmaleimide sensitive fusion protein, an essential protein for vesicular trafficking between ER, Golgi, and PM, does not inhibit the ER to PM transport [74]; two plasma membrane ABCG transporters, Aus1p and Pdr11p, stimulate cholesterol transport from PM to ER [99].

Membranes of virtually any organelle are interconnected through membrane contact sites (MCSs). MCSs connect ER with PM, Golgi, endosomes, lysosomes, mitochondria, and peroxisomes. The MCSs are organized as protein complexes that link the membranes and hold them in the distance about 10-50nm apart and serve as sites for lipid transfer protein- (LTP-) assisted nonvesicle lipid transport. Several LTPs are known to transfer cholesterol between the organelles. The most studied proteins are proteins of ORP family (oxysterol-binding protein- (OSBP-) related proteins) [100–102], STARD family (StAR related lipid transfer domain containing proteins) [100, 103, 104], and sterol carrier protein 2 (SCP-2) [105].

### 3.3. Lipid Transfer Proteins

The oxysterol-binding protein (OSBP) is the founder member of the ORP (OSBP-related protein) family that consists of 12 genes in humans and 7 genes in yeast, Osh1-Osh7. All ORP genes contain lipid binding domain [100]. OSBP is predominately cytosolic protein with minor fraction bound to ER. The ER-bound OSBP forms the MCS between ER and Golgi through binding with vesicle-associated membrane protein-associated protein A (VAP-A) [106]. OSBP binds with high affinity to 25-hydroxycholesterol (K_d_ = 5 nM) and to a number of other oxysterols [100, 107]. The binding to oxysterols leads to translocation of OSBP as well as VAP-A to perinuclear compartments of the cells [106]. OSBP also binds to cholesterol (K_d_ = 70 nM) and phosphatidylinositol-4-phosphate (PI4P) [100]. Overexpression of OSBP in HeLa cells suppresses sterols incorporation into lipid droplets, while the mutant OSBP protein, which is assumed to lose PI4P binding ability, does not interfere with sterols accumulation in LDs.* In vivo* and* in vitro* experiments suggest that OSBP is a PI4P-dependent cholesterol transporter. According to the proposed model, ER-anchored VAP-A at the ER-trans-Golgi MCS binds OSBP, which starts to transfer cholesterol from ER to trans-Golgi, and PI4P in the opposite direction. The gradient of PI4P between two membranes is required, and ER-anchored PI4P phosphatase Sac1 helps in maintaining the PI4P gradient by PI4P degradation while supposedly PI4-kinases continuously regenerate PI4P in the Golgi [106]. Thus, this mechanism requires ATP to maintain PI4P gradient. Similarly, Osh4p transfers ergosterol between ER and trans-Golgi in yeast. Other ORP/Osh proteins are located in the MCSs between ER and PM and other organelles, and most of them are involved in cholesterol transfer [101, 102]. A contribution of ORP6 was investigated in THP-1 macrophages and HepG2 hepatocytes. ORP6 localizes in early endosomes, lysosomes, and the endoplasmic reticulum. Loading of THP-1 cells with cholesterol by acetylated low-density lipoprotein (acLDL) induces ORP6 expression. Knockdown of ORP6 in THP-1 macrophages upregulates mRNA of* SREBF2* and genes induced by* SREBF1/2*,* HMGCR,* and* LDLR*. At the same time ORP6 knockdown inhibits cholesterol efflux to apoA-I and HDL in THP-1 macrophages and inhibits cholesterol efflux to apoA-I in HepG2 hepatocytes. The overexpression of ORP6 results in the opposite effect [1]. Thus, ORP6-dependent cholesterol supply from the intracellular sources significantly affects cholesterol efflux. Knockdown of another ORP protein, ORP8 (or OSBP-related protein 8), in THP-1 macrophages stimulates cholesterol efflux to apoA-I in parallel to an increase of ABCA1 protein [59].

StAR (or STARD1) is the founder protein of the STARD family. It transfers cholesterol from the outer mitochondrial membrane to the inner mitochondrial membrane in steroidogenic tissues for hormone synthesis. The rate of the transfer was estimated as 400 molecules of cholesterol/min per molecule of newly synthesized StAR [108]. StAR overexpression stimulates cholesterol efflux by activating LXR and stimulation of ABCA1 expression. The stimulation of the efflux in cAMP-activated RAW 264.7 macrophages is blocked by sterol 27-hydroxylase inhibitor GW273297x, LXR inhibitor geranylgeranyl pyrophosphate, and ABCA1 inhibitor probucol [49]. Overexpression of StAR stimulates cholesterol efflux to apoA-I in RAW 264.7 macrophages stimulated by agonists of retinoic acid receptor and/or retinoid X receptor all-trans retinoic acid (RA) or 9-cis RA, which activate LXR pathway [50]. STARD3 protein is anchored to late endosomes and together with ER-anchored vesicle-associated membrane protein-associated proteins A and B STARD3 contributes to formation of MCSs between endosomes and ER. Overexpression of STARD3 in HeLa cells, which express STARD3 at very low level, promotes accumulation of cholesterol in LE, while not changing the total cell cholesterol level. Cholesterol depletion of the cells does not prevent cholesterol accumulation in endosomes, while inhibitor of cholesterol synthesis mevinolin prevents the endosomal cholesterol accumulation that indicates on the ER-synthesized cholesterol transport by STARD3. All together, it suggests that STARD3 mediates cholesterol traffic from ER to endosomes, and this route competes with ER to PM traffic. The mechanism of the transfer does not depend on energy [109]. Overexpression of STARD3 in THP-1 cells stimulates cholesterol efflux to apoA-I; however the effect is likely based on the upregulation of ABCA1 mRNA and protein; the efflux to HDL does not change in the STARD3-transfected cells significantly [51]. Another member of STARD family, STARD4 protein, mediates nonvesicular sterol transfer between PM and ERC [98, 110]. In HepG2 hepatocytes expression of STARD4 is induced when cells are incubated in cholesterol-poor conditions. The overall effect of knockdown of the STARD4 on the cell cholesterol level and intracellular distribution depends on the cell type. The knockdown of the STARD4 in HepG2 cells results in the reduction of cholesterol in endoplasmic reticulum and the reduction in the level of cholesteryl esters without significant changes in total cholesterol level [111]. On the contrary, the knockdown of STARD4 in osteosarcoma cells U20S results in an increase in cholesterol level in PM and ERC and in an increase in the level of cholesteryl esters [110].

Sterol carrier protein 2 (SCP-2) binds cholesterol and phospholipids with high affinity. It is localized in peroxisomes and in cytoplasm and involved in cholesterol and phospholipid intracellular transfer. SCP-x, a longer form of SCP-2 that is transcribed from an alternate transcription site, is a peroxisomal 3-ketoacyl-CoA thiolase [105]. The study of SCP-2-deficient fibroblasts from patients with Zellweger syndrome revealed that roughly 50% of ER to PM transport of newly synthesized cholesterol is cytoskeleton- and Golgi-dependent, in contrast to the transport in normal fibroblasts, which does not depend on the cytoskeleton and Golgi. Knockdown of the SCP-2 in normal fibroblasts decreases the fast (10 min) transport from ER to PM by 80%; however the cholesterol is still able to flux to the PM by slower mechanism [112]. Overexpression of SCP-2 in McA-RH7777 rat hepatoma cells sharply increases the rate of the transfer of newly synthesized cholesterol from ER to PM and the amount of newly synthesized cholesterol in the secreted HDL. The overexpression also decreases the rate of CE synthesis without affecting the acyl-CoA:cholesterol acyltransferase and neutral cholesterol ester hydrolase activities measured* in vitro*. In addition, it does not affect the transport of LDL-derived cholesterol to the PM [113]. Because the SCP-2 overexpression stimulates cholesterol transport from ER to PM and stimulates secretion of cholesterol with HDL in hepatoma cells, the SCP-2 overexpression in L-cell fibroblasts inhibits cholesterol efflux to HDL at HDL concentration below 100 *μ*g/ml [63]. Possible clue to explanation of negative effect of SCP-2 on cholesterol efflux is the inverse relationship between the expression of SCP-2 and liver fatty acid-binding protein (L-FABP aka FABP1). L-FABP is a hepatic cytosolic protein that binds long-chain fatty acids and other hydrophobic molecules including cholesterol. L-FABP protein level is twice higher in hepatocytes from SCP-2/SCP-x knockout compared to hepatocytes from normal mouse. Cholesterol efflux to HDL in hepatocytes from SCP-2/SCP-x knockout mouse is 35% higher than in normal mouse hepatocytes. However, cholesterol efflux in hepatocytes from L-FABP^−/−^/SCP-2/SCP-x^−/−^ mouse is decreased compared to hepatocytes from WT mouse that indicates positive effect of L-FABP on the cholesterol efflux [114]. Thus, overexpression of SCP-2 might possibly repress the expression of L-FABP and inhibit the efflux. Besides cytoplasmic localization, SCP-2 and L-FABP are found on the PM in close proximity to SR-BI. Some data suggest that L-FABP promotes uptake of HDL cholesterol by SR-BI in mouse hepatocytes [115].

### 3.4. Caveolae Cholesterol Is Actively Consumed in Cholesterol Efflux from the Cells

Caveolae are cholesterol-rich microdomains of PM formed by caveolin protein that are likely assembled in Golgi and transported to the PM. Both the newly synthesized and derived from LDL cholesterol pools first appear in caveolae and then they spread to non-caveolae areas of PM in human fibroblasts [116, 117]. In fibroblasts, at least 70% of cholesterol from LDL is quickly transported to caveolae domains of PM by a Golgi-dependent pathway. This path does not depend on the functional cytoskeleton. The rest of cholesterol from LDL is transported from lysosomes to the ER. This path is not Golgi-dependent and is inhibited by the disruption of actin filaments that suggests vesicular transport [4]. In yeast, contrary, newly synthesized ergosterol, the major yeast sterol, first appears mostly in non-raft fraction of PM. Then it equilibrates with raft sterol in about 1 h.

The caveolar cholesterol is the primary PM cholesterol that is effluxed by the fibroblasts to plasma or HDL [116]. The nascent HDL produced by cholesterol efflux to apoA-I by human embryonic kidney (HEK) 293 cells that express ABCA1 or mouse BMDMs are closer in cholesterol:PC and SM:PC ratio to rafts than to the PM fraction [118]. Caveolae formations start with expression of integral membrane proteins caveolin-1 or caveolin -2 in ER followed by their oligomerization (7-14 molecules of caveolin) and COPII-dependent transportation to the Golgi. The oligomer size in the Golgi increases to 18-25 molecules of caveolin and the oligomer-associated membrane saturates with cholesterol molecules. Then the cholesterol-rich complex is transported from trans-Golgi network to PM by four phosphate-adapter protein (FAPP1, FAPP2) secretory vesicles. Phosphatidylinositol-4-phosphate (PI4P), the prevalent phosphoinositide species in Golgi membrane, and small GTPases ARF1 play the essential role in the formation of these secretory vesicles [119]. Caveolin family consists of three genes. They share the property of insertion into the lipid membrane, the generation of oligomers with the formation of specific lipid rafts caveolae, which are cholesterol-enriched flask-shaped membrane invaginations of 50-100 nm in size [119]. Caveolins bind cholesterol and make specific pool of cholesterol on the PM [120, 121]. Caveolin-1 interacts with SCP-2 protein in caveolae in PM as well in cytosolic caveolar vesicles and caveolin/chaperone complexes of L-cells fibroblasts [121]. Cholesterol efflux to plasma is increased in fibroblasts transfected by caveolin-1 [38]; however the effect of the caveolin-1 overexpression on the efflux is not observed in CHOP cells [36]. Caveolins are not expressed in RAW 264.7 cells and caveolin-1 expression stimulates cholesterol efflux to HDL but not to apoA-I or to plasma [17, 36, 122]. In THP-1-derived macrophages, the knockdown of caveolin-1 by antisense DNA inhibits cholesterol efflux to apoA-I with minimal effect on the efflux of PL [37]. A reduced cholesterol efflux to serum is observed in Cos-1 cells transfected with dominant-negative mutant of caveolin-1 that is not transported to the PM [123]. Transfection to express caveolin-1 in HepG2, hepatocyte cell line that does not express caveolin-1, results in an increased efflux to human plasma or apoA-I but not to cyclodextrin [36].

Contrary to stimulatory effect of caveolin-1 on cholesterol efflux observed in the most other cell cultures, mouse embryonic fibroblasts from caveolin-1 knockout mouse have an increased efflux to apoA-I compared to the cells from wild type animal. Induction of ABCA1 gene expression by LXR agonist increases this difference. Caveolin-1 disruption partly prevents apoA-I from its internalization into the cells and degradation. It is proposed that high curvature of the PM at the neck of caveolae is an attractive site for apoA-I binding to PM and the bound apoA-I is subjected to uptake and degradation [124].

## 4. Molecular Mechanisms of Cholesterol Efflux

**Figure 2:**
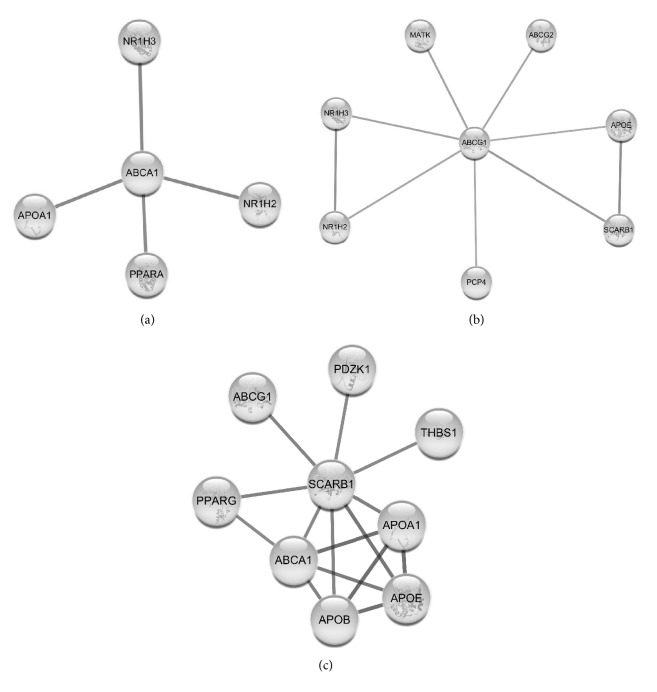
Protein-protein interactions involved in cholesterol efflux by cholesterol transporters. Data for human ABCA1 (a), ABCG1 (b), and SR-B1 (SCARB1) (c) molecules were imported from STRING database with Cytoscape STRING plugin. Confidence cutoff for interactions was chosen as 0.95, 0.7, and 0.8, respectively. (a) apoA-I is the major protein component of HDL; PPARA is a nuclear receptor, transcription factor, key regulator of lipid metabolism; NR1H2 is an oxysterols receptor LXR-beta–nuclear receptor that regulates cholesterol uptake; NR1H3 is an oxysterols receptor LXR-alpha-nuclear receptor that regulates homeostasis and cholesterol uptake. (b) NR1H3 and NR1H2 are mentioned above; MATK is a megakaryocyte-associated tyrosine-protein kinase that could play a significant role in the signal transduction of hematopoietic cells. PCP4 is a Purkinje cell protein 4 that plays an important role in synaptic plasticity,* regulating calmodulin function;* ABCG2 is an urate exporter that is able to mediate the export of protoporhyrin IX and implicated in the efflux of numerous drugs and xenobiotics; apoE is an apolipoprotein that mediates the binding, internalization, and catabolism of lipoprotein particles; SR-B1 (SCARB1) is a receptor for different ligands, receptor for HDL. (c) apoA-I and apoE are mentioned above; ABCA1 and ABCG1 play a role in HDL metabolism; apoB is a major protein component of chylomicrons, VLDL, and LDL; PPARG is a nuclear receptor that controls the peroxisomal beta-oxidation pathway of fatty acids and regulates adipocyte differentiation and glucose homeostasis; PDZK1 is a PDZ domain containing scaffolding protein; THBS1 is a thrombospondin-1, adhesive glycoprotein, that mediates cell-to-cell and cell-to-matrix interactions, binds heparin.

Four major pathways mediate cholesterol efflux from cell plasma membrane and the contribution of the particular pathway varies depending on the cell type and extracellular acceptor nature (Table 2). These pathways include two ABC-transporters, ABCA1 and ABCG1, along with SR-BI and passive aqueous diffusion [125]. All the proteins mediating efflux from PM to extracellular acceptor are also involved in the intracellular cholesterol traffic and cholesterol distribution between various intracellular pools. The aqueous diffusion occurs for any cells; however, its contribution to the total cholesterol efflux for most of the type of cells is relatively small. All three proteins interact with other proteins at cholesterol efflux and the most significant pairs are given on Figure 2.

### 4.1. Aqueous Diffusion

Cholesterol can diffuse between membranes of cells, liposomes, and emulsions through the aqueous phase. The efficiency of nondirectional diffusional transfer of cholesterol is determined by the cholesterol capacity of the membrane and the kinetic factors—the rate of desorption and the concentration gradient. Cholesterol molecules desorbed from membranes are absorbed by various acceptors: plasma lipoproteins, plasma albumin and globulins, liposomes, and microemulsions, as well as specific molecules such as cyclodextrins [126].

When measuring cholesterol exchange rate between donor (6.25 mg/ml) and acceptor (0.4 - 9.0 mg/ml) single layer liposomes consisting of 20% cholesterol and 80% phospholipids that were separated by a dialysis membrane, the kinetics corresponded to a first-order reaction, which is characteristic of cholesterol transfer by the mechanism of free diffusion ((1) and (2)). The rate-limiting step in the exchange was the rate of cholesterol desorption [127]. However, this behavior is characteristic only for low concentrations of vesicles (< 3 mM). The kinetics of the second order, characteristic for the collision mechanism, is observed at high cholesterol concentrations [128] ((3)-(4)):

(1) D-Ch⟷D+Ch,

(2) A+Ch⟷A-Ch,

(3) D-Ch+A⟷D-Ch-A,

(4) D-Ch-A⟷D+A-Ch,

where A is the acceptor, Ch is the cholesterol, and D is the donor.

When measuring the rate of intermembrane cholesterol transfer, the following deviations from the diffusion mechanism were observed: (1) the rate of cholesterol transfer from erythrocytes to acceptors was inversely related to the size of the acceptor; (2) the rate of cholesterol transfer from erythrocytes to the erythrocyte ghosts increased with the addition of plasma, while the opposite effect could be expected due to competition between ghosts and plasma components that act as cholesterol acceptors; (3) the rate of transfer decreased upon the dilution of the mixture of erythrocytes and ghosts but did not obey the second-order kinetics; (4) cholesterol in the membranes of the bovine retina rod cells is not in equilibrium with the cholesterol of the plasma and its content increased after incubation with plasma; (5) the deviation of transfer kinetics from the diffusion model can not be explained by the presence of a non-stirred layer, since the transfer rate of lysolecithin was three orders of magnitude higher. The authors suggested a combined model of the primary activation of cholesterol (Ch′) in the donor membrane in the first-order kinetics followed by the formation of the donor-acceptor complex in second-order kinetics [129] ((5)-(7)):

(5) D-Ch↔D-Ch′,

(6) D-Ch′+A↔D-Ch′-A,

(7) D-Ch′-A↔D+Ch′-A.

A similar model was proposed for the transfer of cholesterol from erythrocytes to HDL_2_, HDL_3_, or LDL, when the cholesterol exchange rate is nonlinearly dependent on the concentration of lipoproteins at their low concentrations and reaches a plateau at high concentrations of lipoproteins. In this model, the cholesterol transfer from erythrocytes is determined by the collision mechanism at low concentrations of acceptors, while the diffusion of cholesterol in the erythrocyte membrane to the specific sites of lipoprotein adsorption becomes rate limiting at high concentrations of acceptors [130].

However, the transfer of cholesterol between vesicles and reconstituted HDL (rHDL) was not consistent with aqueous diffusion or collision mechanisms. It was suggested that apoA-I of rHDL interacts with vesicles, which facilitates the transfer of cholesterol, and the interaction depends on the conformation of the apolipoprotein. This model was consistent with the data on the transfer of cholesterol from vesicles to rHDL with different amounts of apoA-I and explained the effect of the composition of rHDL and vesicles on the rate of cholesterol transfer [131]. Opposite, the collision mechanism at cholesterol exchange between vesicles and rHDL (apoA-I-containing nanodiscs) was discarded in the work of Matsuzaki et al. The authors postulated the importance of the diffusion mechanism with cholesterol dissociation from the vesicles as a rate-limiting step of the cholesterol transfer. Interestingly, the rate of dissociation of cholesterol from rHDL bilayer was higher than the rate of dissociation from the bilayer of liposomes with similar composition [132]. The authors suggested that it might be explained by denser bilayer packing in rHDL. Another reason may be the appearance of “active” cholesterol in the nanodiscs due to the heterogeneity of the distribution of cholesterol in discoidal lipoproteins. Such heterogeneity we found in the reconstituted particles containing various apolipoproteins [133].

### 4.2. ABCA1

ABCA1 is localized in the plasma membrane and in late and early endosomes [134]. The LE-located ABCA1 seems to assist in efflux of LE pool of cholesterol [135]. It was reported that overexpression of ABCA1 prevents the accumulation of cholesterol in LE and lysosomes in NPC1-deficient, but not NPC2-deficient cells [136]. Another study demonstrated that ABCA1 participates in the cholesterol traffic from PM to ER. The reduction of the ability of the membrane to retain cholesterol after treatment by sphingomyelinase leads to rapid flow of PM cholesterol inside the cell, its esterification, and inhibition of cholesterol synthesis [72]. This PM to ER flow is mediated by ABCA1 by about 50% in mouse embryonic fibroblasts. ABCA1 mutant lacking ATPase activity is unable to provide the PM to ER cholesterol transport [137]. Bioinformatics analysis of functional and physical interactions of ABCA1 reveals apoA-I and several nuclear receptors (acting as transcriptional factors) as major partners/modulators of ABCA1 (Figure 2(a)).

ABCA1 structure includes two hydrophobic transmembrane domains, each containing six *α*-helices, and two hydrophilic domains, called nucleotide-binding folds [138]. ATP hydrolysis in two hydrophilic domains of ABCA1 results in a change in the protein conformation accompanied by the transfer of the transported molecule to the outer part of the membrane. ABCA1 transports phosphatidylcholine, phosphatidylinositol (4,5) bis-phosphate (PIP2) [44], and less efficiently phosphatidylserine and sphingomyelin [139]. Recently we described the existence of two types of putative cholesterol-binding motifs in ABCA1 and their involvement into binding of cholesterol molecules differently immersed in lipid bilayer (Dergunov et al., 2018* submitted*).

An increase in the cholesterol content in the cell, firstly, inhibits ubiquitination and subsequent degradation of ABCA1, thereby increasing the level of the ABCA1 protein [140] and, secondly, induces the expression of genes involved in cholesterol efflux [68]. ABCA1 molecules undergo palmitoylation of cysteine residues 3, 23, 1110, and 1111. In the absence of these modifications, the transporter molecules remain inside the cell. The absence of any of these palmitoylation reduces cholesterol efflux from the cells [141]. Cholesterol efflux mediated by ABCA1 results in the formation of discoidal HDL, containing two, three, or four molecules of apoA-I per particle. Nascent HDLs are heterogeneous in size and composition and contain the main classes of lipids present in plasma membrane. In addition to discoidal HDL, ABCA1-mediated efflux generates lipid-poor apoA-I with one molecule of apolipoprotein [142, 143]. Cholesterol of the plasma membrane can be replenished from endosomal compartments in the minute range [144]. Importantly, a direct relation between ABCA1-mediated cellular cholesterol efflux and arterial-wall thickness exists that suggests the inhibition of atherosclerosis progression by efflux increase before the manifestation of symptomatic cardiovascular disease [145]. However, some controversy exists on the contribution of both common and rare ABCA1 variants and levels of HDL cholesterol to risk of ischemic heart disease in the general population [146, 147]. Interestingly, stimulation of macrophage mitochondrial ATP production resulted in the increase of ABCA1 expression and cholesterol efflux with a concomitant decrease in aortic sinus lesion area in atherosclerosis-prone mice, despite no changes in HDL cholesterol [148]. Besides, the protective effect of ABCA1 pathway activation in reactive astrocytes at ischemic stroke has been suggested [149].

The nature of the molecular interaction between various cholesterol acceptors and ABCA1 is controversial, and two alternative models suggesting a direct protein-protein interaction or indirect association have been proposed. According to the first model, the apoA-I and ABCA1 molecules interact [150, 151]. In the second two-site model, besides the direct ABCA1-apoA-I interaction, the existence of much more pronounced association of the apolipoprotein with the lipid phase near the transporter molecule is suggested [152]. Both models recognize the significant contribution of the interaction of apoA-I and membrane lipids in the ABCA1-mediated cholesterol efflux. ABCA1 translocates phospholipids to the exofacial leaflet of the plasma membrane bilayer that creates membrane tension. Apolipoprotein-lipid interaction results in the decrease of this tension [139]. The introduction of the amphipathic helix of apoA-I into protrusion on the plasma membrane [153], similar to the solubilization by apolipoprotein of multilayer vesicles [143, 154], leads to the dissociation of cholesterol and phospholipids in apolipoprotein-lipid complexes from the plasma membrane. Note that the complexes primarily include cholesterol, not contained in the rafts [155].

According to the model of the direct interaction between the apolipoprotein and the transporter, the mechanism of the ABCA1-mediated efflux of cholesterol includes the following steps: (1) diffusion of the transporter in a membrane and ATP-dependent lipid flopping; (2) dimerization of the transporter and fixation of the dimer in the membrane involving the cytoskeleton; (3) the interaction of lipid-free apoA-I with the dimer, followed by apolipoprotein-lipid interaction, dissociation of the apolipoprotein-transporter complex, and the release of the lipid-laden apoA-I into the extracellular environment; (4) dissociation of the dimer with closing of the ABCA1 cycle [156]. The alternative model questions the exclusiveness of the direct interaction of apoA-I and ABCA1. Experiments on human fibroblasts have shown that most (90%) of the apoA-I molecules bind to the HCBS (high capacity binding site), and not to ABCA1. Interestingly, HCBS is not a part of the lipid rafts. HCBS mainly consists of phosphatidylcholine, and not sphingomyelin, and is not associated with caveolin-1. PIP2 plays significant role in the binding of apoA-I to the membrane, and PIP2 is transferred to the exofacial leaflet of plasma membrane by the floppase activity of ABCA1 [44]. The disruption of the interaction of the apolipoprotein with HCBS significantly reduced cholesterol efflux on apoA-I. According to this model, ABCA1 generates HCBS, and the direct interaction of apoA-I with ABCA1 could stabilize the structure of ABCA1 [152, 157]. Wang et al. suggested the existence in ABCA1 structure a nonspecific low-affinity site of binding with apoA-I. This site serves as a chaperone in the unfolding of the N-terminus of the apolipoprotein, which is an important process in the formation of HDL [158].

### 4.3. ABCG1

ABCG1 is another, along with ABCA1, ATP-binding cassette transporter that participates in cholesterol efflux in macrophages (Table 2). There is some inconsistency in the data on ABCG1 localization. In a recent study in CHO and HeLa cell lines that were transfected to stably express ABCG1 fused with Myc tag or green fluorescent protein (GFP), the transporter was distributed between PM and ER pools. The PM pool increased after loading the cells with cholesterol [159]. In another study of ABCG1-GFP fusion, expressed in HeLa cells, the transporter was observed in PM and late endosomes [160]. However, another group, using several approaches, found that intracellular endosomes are the only sites of ABCG1 localization, and ABCG1 is not observed in PM neither in murine peritoneal macrophages, nor in CHO, Cos-7, and HEK293 cells transfected with untagged or FLAG-tagged ABCG1 [161, 162].

Structurally, ABCG1 contains a hydrophobic transmembrane domain of six *α*-helices and a hydrophilic nucleotide-binding folds domain. It resembles half of ABCA1 transporter and is called sometimes the half-transporter [163]. ABCG1 is palmitoylated at positions 26, 150, 311, 390, and 402. The palmitoylation at Cys311 is critical for the localization of ABCG1 at the plasma membrane and for ABCG1-dependent cholesterol efflux, while palmitoylation at other positions is not essential for these processes [164]. In addition, tyrosine Y667 of cholesterol-binding motif of ABCG1 is functionally important: the substitution of this tyrosine reduced the efflux [165]. Interestingly, ABCG1, in addition to cholesterol, transports sphingomyelin, and different structural motifs are responsible for binding of these lipids. It was suggested that sphingomyelin stimulates cholesterol efflux by ABCG1 via activation of its ATPase activity [166]. The cholesterol-binding motif, which is important for the cholesterol efflux activity of ABCG1, was also found in half-transporters ABCG4 and ABCG8 that mediate cholesterol efflux [165]. We predicted the presence of two types of cholesterol-binding motifs in the ABCG1 structure that mediate the transport of different pools of cholesterol (Dergunov et al., 2018* submitted*).

ABCG1 expression results in the increase of the rate of cholesterol desorption and in the increase of cholesterol pool available for both efflux and esterification [167, 168]. The major partners/modulators of ABCG1 that were determined by bioinformatics analysis are presented on Figure 2(b). The expression of ABCG1 is associated with an increase in the efflux of cholesterol on HDL_2_, HDL_3_, LDL, SUV, and rHDL, but not on lipid-free apoA-I and does not affect cholesterol influx [167, 169]. Despite the massive lipid accumulation in hepatocytes and in macrophages in mice with targeted disruption of* Abcg1* gene and the promotion of cholesterol efflux to HDL in human ABCG1-transgenic mice, the plasma lipid level did not change in both cases [169]. Moreover, ABCG1 in the arterial wall might possess a proatherogenic effect independently of any modulation of HDL-C level [170]. ABCG1 is not expected to attenuate foam cell formation in early atherosclerosis lesions in humans [171] but could protect from atherosclerosis by preserving vascular endothelium from dietary cholesterol-induced dysfunction [172]. There is no specific binding of the transporter to the lipoprotein in ABCG1-mediated efflux of cholesterol. For SUV, LDL, and rHDL discs as cholesterol acceptors, the kinetics of the ABCG1-mediated efflux corresponds to the aqueous diffusion mechanism, and ABCG1 increases the pool of active cholesterol available for efflux. The kinetics of efflux with HDL_2_ and HDL_3_ deviates from the kinetics for diffusion, presumably due to the shielding by apolipoproteins of cholesterol-accepting phospholipid patches. In experiments with BHK cells, the size of the pool of cholesterol available for efflux on HDL_3_ as an acceptor was about 20% of total cell cholesterol. The expression of ABCG1 increased the pool size by 6-12% [167]. Neufeld et al. also observed the increase of the pool of active cholesterol available for efflux. Both PM- and late endosome-localized ABCG1 contributed to the stimulation of cholesterol efflux. LE-localized ABCG1 stimulated cholesterol transfer from the LE to PM, perhaps by direct interaction between LE and PM membranes, and/or in a process, mediated by nonvesicular cytosolic cholesterol carrier proteins such as OSBP [160].

### 4.4. SR-B1

Scavenger receptor class B type 1 (SR-B1) belongs to the superfamily of CD36 scavenger receptors. SR-B1 is highly expressed in the liver, where it participates in RCT [173], in steroidogenic tissues (adrenal gland and ovary) and mammary gland during lactation [174]. According to the bioinformatics analysis of physical and functional interactions, the most important SR-B1 protein partners are apolipoproteins and proteins involved in HDL metabolism, nuclear receptor PPAR*γ*, a scaffold protein PDZ domain containing 1 (PDZK1) that controls SR-B1 level in liver [175, 176], and thrombospondin-1 (THBS1), a subunit of disulfide-linked homotrimeric protein that binds components of extracellular matrix and mediates cell-to-cell interactions (Figure 2(c)). The expression of THBS1 is downregulated by HMG-CoA reductase inhibitor lovastatin [177].

SR-B1 molecule contains two hydrophobic domains and an extracellular glycosylated domain [178]. The cytoplasmic C-terminal domain of SR-B1 binds to PDZ domains of PDZK1 protein [179]. SR-B1, being an HDL receptor, selectively transfers cholesteryl ester molecules contained in HDL into a cell without endocytosis and degradation of HDL particle [180]. In addition to HDL, the receptor exhibits broad ligand specificity for LDL, ox-LDL, and VLDL [181]. As the diameter of HDL increases, the maximum binding value *B*_max_ increases, and the value of the dissociation constant *K*_d_ decreases; that is, the large HDL are the best ligands for SR-B1 [182]. Nevertheless, HDL binding to SR-B1 is about 150-fold lower than the binding of LDL to LDL receptor. The weaker binding and the faster dissociation of HDL from SR-B1 can explain why HDL does not undergo endocytosis. The isotherm of binding of HDL to SR-B1 on the cell surface most corresponds to the presence in SR-B1 of one high affinity and one low-affinity binding sites. The rate of cholesterol transfer from HDL increases proportionally with their CE content. The current model of SR-B1 action suggests CE transfer between HDL and PM through the hydrophobic channel along the concentration gradient [183]. This mechanism can be viewed as a facilitated diffusion. It is assumed that the transfer of cholesterol similarly occurs through the hydrophobic channel. However, SR-B1 also redistributes the membrane pools of cholesterol, as evidenced by the increase in the amount of cholesterol available for oxidation with cholesterol oxidase [184]. Perhaps the cholesterol-binding motifs predicted by us in the structure of SR-B1 (Dergunov et al., 2018* submitted*) are responsible for the redistribution of cholesterol in the membrane.

It is important that the influx of the CE correlates with the binding of HDL, while the efflux of cholesterol continues to increase after the receptor is saturated with the lipoprotein. Perhaps this is due to the SR-B1-induced redistribution of the cholesterol membrane pools in favor of the efflux by the diffusion mechanism or because of collisions [182]. Some artificial chimeric receptors with partial substitution of the SR-B1 sequence by the sequence of another SR-B class receptor CD36 retain the ability to efflux cholesterol even with significantly decreased binding of HDL. These chimeric receptors also retained the ability of SR-B1 to increase the cholesterol pool accessible to cholesterol oxidase [185]. Functional analysis of SR-B1 mutants shows that various activities of SR-B1, such as CE influx, cholesterol efflux, redistribution of membrane pools of cholesterol, and HDL binding, are partly independent due to the presence of mutations that affect some of the activities and do not affect others [185–188].

## 5. Conclusion

The gradient of cholesterol concentration between plasma membrane and the membranes of intracellular organelles is maintained by constant redistribution of the cholesterol in energy-dependent vesicular traffic and energy-independent transport by lipid transfer proteins. Vesicular traffic plays just a minor role in the cholesterol transfer from the site of synthesis (ER) to PM; however it plays the central role in endocytosis of LDL-delivered cholesterol. The fractions of fast- and slow-exchanging pools of cholesterol are determined by the membrane lipid composition and the kinetics of the association and dissociation of the cholesterol complexes with other membrane lipids, such as in lipid rafts, which are stable in some range of the cholesterol/phospholipid ratio in the membrane. Just partly exposed into the aqueous phase cholesterol molecules that are not incorporated to lipid complexes are available to the fast escape from the membrane, which is the rate-limiting event in the aqueous diffusion pathway in cholesterol efflux. The facilitated diffusion along the concentration gradient through the hydrophobic tunnel occurs in the cholesterol efflux mediated by SR-B1. ABCG1-mediated cholesterol efflux is supposedly determined by the increase of the pool of active cholesterol that is available for the transfer from the membrane to lipoproteins by aqueous diffusion. Active cholesterol may originate from the specific cholesterol-binding motifs in ABCG1 structure at membrane interface. ABCA1-mediated efflux occurs due to the floppase activity of the transporter powered by the hydrolysis of ATP. ApoA-I binding to the cell surface is essential in the ABCA1-mediated cholesterol efflux; however it is not clear yet whether apoA-I binds to ABCA1 or to the lipid phase of plasma membrane. A number of intracellular processes determine the availability of the cholesterol for the efflux to extracellular acceptors.

## Figures and Tables

**Table 1:** Genes that participate in the intracellular cholesterol traffic and cholesterol homeostasis and affect the efflux of cholesterol to extracellular acceptors. Stimulation of cholesterol efflux: observed as inhibition for knockdown/knockout or stimulation for overexpression; inhibition of cholesterol efflux: observed as stimulation for knockdown/knockout or inhibition for overexpression.

Gene	Description	Cell^(a)^	Acceptor	Reference
	*Stimulation of cholesterol efflux*			

ABCA1 (ATP-binding cassette transporter A1)	Mediates cholesterol efflux	MPM, THP-1, Huh7.5 (hepatoma cells), human foreskin fibroblasts	apoA-I	[29–31]
		BHK expressing ABCA1	M*β*-CD	[32]
ABCG1 (ATP-binding cassette transporter G1)	Mediates cholesterol efflux	J774, THP-1	HDL	[33]
^(b)^ ARF6 (ADP-ribosylation factor 6)	A GTP-binding protein of the Ras family; regulates endocytosis and endocytic recycling of plasma membrane proteins	Cholesterol-depleted RAW 264.7	apoA-I	[15]
BIG1 (Brefeldin A-inhibited guanine nucleotide-exchange protein 1)	Mediates intracellular vesicular trafficking	HepG2	apoA-I	[34]
CANX (Calnexin)	An integral ER membrane calcium-binding lectin-like chaperone	HeLa expressing ABCA1, THP-1	apoA-I	[35]
CAV1 (Caveolin-1)	A scaffolding protein; binds cholesterol; required to formation of caveolae	RAW 264.7	HDL	[17, 36]
		HepG2, THP-1	apoA-I	[36, 37]
		HepG2, fibroblasts	plasma	[36, 38]
^(b)^ CDC42 (Cell Division Cycle 42)	A GTPase of the Rho family; regulates endocytosis and endocytic recycling of plasma membrane proteins	Cholesterol-depleted RAW 264.7	apoA-I	[15]
CTH (Cystathionine *γ*-lyase)	Produces endogenous H_2_S, induces ABCA1 expression by activation of PI3K/AKT pathway	THP-1	apoA-I, HDL	[39]
CTSD (Cathepsin D)	A lysosomal proteinase, activated by ceramide	J774, CHO	apoA-I	[40]
^(b)^ DNM2 (Dynamin-2)	A GTPase; regulates endocytosis and endocytic recycling of plasma membrane proteins	Cholesterol-depleted RAW 264.7	apoA-I	[15]
HNF4A (Hepatocyte nuclear factor 4*α*)	A nuclear transcription factor; required for early liver development and expression of many liver-specific genes; induces expression of ABCA1	JHH-5 cells (human hepatoma)	apoA-I	[41]
ELAVL1 (ELAV-like protein 1 aka Human antigen R aka HuR)	RNA binding protein; modulates the stability and translational efficiency of mRNAs; promote ABCA1 translation	THP-1	apoA-I	[42]
NPC1 (Niemann-Pick disease, type C1)	Mediates cholesterol trafficking from late endosomes and lysosomes	MPM	HDL2	[16]
OSBPL6 (Oxysterol-binding protein-like 6; aka ORP6)	Participates in cholesterol trafficking between endosomes and endoplasmic reticulum; a target for miR-33 and miR-27b	THP-1, HepG2	apoA-I, HDL (THP-1, n.d. for HepG2)	[1]
PIM1 (Proto-Oncogene, Serine/Threonine Kinase)	Serine/threonine-protein kinase, phosphorylates ABCA1 that retards its degradation	HepG2	apoA-I	[43]
PIP5K1A (Phosphatidylinositiol-5-phosphate 4-kinase *α*)	Synthesizes phosphatidylinositol 4,5-bisphosphate (PI[4,5]P2) from phosphatidylinositol 4-phosphate (PI4P)	RAW264.7, HEK293 expressing ABCA1	apoA-I	[44]
PPARG (Peroxisome proliferator-activated receptor *γ*)	A nuclear receptor that regulates lipid and glucose metabolism; induces ABCG1 expression	mBMDM	apoA-I	[45]
RAB8A (Rab8)	A small GTPase of the Ras family; participate in cytoskeletal organization and membrane trafficking; induces ABCA1	hPBMC	apoA-I	[46]
^(b)^ SCD (Stearoyl-coenzyme A desaturase 1)	Converts saturated stearic acid (18:0) to monounsaturated oleic acid (18:1)	RAW 264.7	HDL, HDL2, HDL3, mouse plasma	[47]
SNTB1 (*β*1-syntrophin)	A structural protein containing PDZ domain that anchors membrane proteins to cytoskeleton; interacts with ABCA1 and promotes its expression	mBMDM, primary human fibroblasts	apoA-I	[48]
STAR (Steroidogenic acute regulatory protein, StAR)	Transports cholesterol to the inner mitochondrial membrane to sterol 27-hydroxylase. Activates LXRs and promotes expression of ABCA1.	RAW 264.7	apoA-I	[49, 50]
STARD3 (StAR related lipid transfer domain containing 3)	Endosomal cholesterol transporter; promotes expression of ABCA1	THP-1	apoA-I	[51]
TSPO (Translocator protein)	A small (18 kDa) integral membrane mitochondrial trafficking protein; participates in cholesterol transport, interacts with StAR; promotes expression of ABCA1	THP-1	apoA-I, HDL, serum	[52]
US28	A cytomegalovirus (HCMV) protein; activates CDC42; responsible for restructuring of lipid rafts in the host cells	human foreskin fibroblasts	apoA-I	[31]
UTRN (Utrophin)	A scaffold protein that interacts with syntrophins and actin cytoskeleton; promotes ABCA1 expression	primary human fibroblasts	apoA-I	[48]

	*Inhibition of cholesterol efflux*			

ACSL1 (Acyl-CoA synthetase 1)	A key enzyme mediating synthesis of fatty acyl-CoA esters in macrophages	J774	apoA-I	[53]
ADIPOR2 (Adiponectin receptor 2)	Adiponectin regulates fatty acid oxidation and other metabolic processes. The receptor suppresses expression of ABCA1, ABCG1	THP-1	apoA-I, HDL	[54]
^(b)^ ARF6 (ADP-ribosylation factor 6)	A GTP-binding protein of the Ras family; regulates endocytosis and endocytic recycling of plasma membrane proteins	RAW 264.7	apoA-I, mBMDM	[15]
^(b)^ CDC42 (Cell Division Cycle 42)	A GTPase of the Rho family; regulates endocytosis and endocytic recycling of plasma membrane proteins	RAW 264.7	apoA-I	[15]
^(b)^ DNM2 (Dynamin-2)	A GTPase; regulates endocytosis and endocytic recycling of plasma membrane proteins	RAW 264.7	apoA-I	[15]
ENTPD1 (Ectonucleoside triphosphate diphosphohydrolase-1 aka CD39)	Anchored to plasma membrane and hydrolysis extracellular ATP, which is increased when ABCA1 is highly expressed	RAW 264.7, BHK expressing ABCA1	apoA-I	[55]
IRAK1 (Interleukin-1 receptor-associated kinase-1)	Participates in signaling via TLR (Toll-like receptors)/IL-1R (interleukin 1 receptor)	THP-1	apoA-I, HDL	[56]
LCK (Lymphocyte-specific protein tyrosine kinase)	A tyrosine kinase of Src family; participates in T-cell receptor signaling	Jurkat cells	apoA-I	[57]
LPL (Lipoprotein lipase)	A secreted enzyme facilitating the hydrolysis of triglycerides in chylomicrons	THP-1	apoA-I	[58]
OSBPL8 (OSBP-related protein 8 ala ORP8)	An endoplasmic reticulum protein that binds 25-hydroxycholesterol and other lipid molecules and transfer them between ER and PM; inhibits ABCA1 expression	THP-1	apoA-I	[59]
PLIN2 (Perilipin 2 aka Adipophilin)	Protein, associated with intracellular lipid droplets; a marker of lipid accumulation	THP-1	apoA-I	[60]
PTX3 (Pentraxin 3)	A member of the pentraxin family together with short pentraxins, such as C-reactive protein and serum amyloid P component; releases in response to inflammation; inhibits ABCA1 expression	THP-1	apoA-I	[61]
ROCK2 (Rho Associated Coiled-Coil Containing Protein Kinase 2)	Serine-threonine protein kinase; involved in the regulation of the actin cytoskeleton	mBMDM	apoA-I	[45]
^(b)^ SCD1 (Stearoyl-coenzyme A desaturase 1)	converts saturated stearic acid (18:0) to monounsaturated oleic acid (18:1)	THP-1	no acceptor	[62]
SCP2 (Sterol carrier protein-2)	Mediates cholesterol and phospholipid intracellular trafficking	L-cell fibroblasts	HDL	[63]
SPTLC1 (Serine palmitoyltransferase long chain base subunit 1)	The key enzyme in sphingolipid biosynthesis; blocks the exit of ABCA1 from the endoplasmic reticulum	primary human fibroblasts, HEK 293-EBNA-T cells expressing ABCA1	apoA-I	[64]
SREBP2 (Sterol-responsive element-binding protein 2)	A transcription factor regulating cholesterol synthesis and uptake; N-terminus is an active form of SREBP2; inhibits ABCA1 expression	HUVEC	apoA-I	[65]
TMEM55B (Transmembrane Protein 55B)	A specific PIP2 phosphatase that converts PIP2 (phosphatidylinositol (4,5) bis-phosphate) to PI5P phosphatidylinositol 5-phosphate)	RAW264.7	apoA-I	[44]
TNFRSF25 (TNF receptor superfamily member 25 aka Death receptor 3)	A cell surface receptor of TNF-like protein 1A (TL1A); highly expressed in foam cells in atherosclerotic plaques; TL1A inhibits expression of ABCA1 and ABCG1	THP-1, hPBMC	apoA-I	[66]
ZNF202 (Zinc finger protein 202)	a transcriptional repressor of ABCA1 and ABCG1	RAW 264.7	HDL3	[67]

^(a)^In some cases, cells were treated to differentiate to macrophages (e.g., by PMA (phorbol 12-myristate 13-acetate) or colony-stimulating factor GM-CSF), to induce expression of ABCA1 (e.g., by cpt-cAMP, TO-901317, or 22-OH+9cRA), and transformed to foam cells (e.g., by acLDL).

^(b)^denotes that the gene has both stimulating and inhibiting effect depending on the type of cells and/or cholesterol acceptor.

hPBMC: human peripheral blood mononuclear cells; HUVEC: human umbilical vein endothelial cells; mBMDM: mouse bone-marrow-derived macrophage; MPM: mouse peritoneal macrophage; M*β*-CD: methyl *β* cyclodextrin.

**Table 2:** The contribution of specific pathways in cholesterol efflux. The estimations are from the tables, texts, and graphs in the cited papers. Some values do not give a total of 100%, probably because of the rounding of the data in the tables. Efflux values are given as a percentage of total cell cholesterol and the contribution of pathways in the original studies was estimated using inhibitors of ABCA1 and SR-BI (probucol and BLT-1), if other is not mentioned.

Cells	Acceptor	Efflux, %	Contribution of the pathway to efflux, %					Reference
			Uninhibited^(a)^	Diffusion	ABCA1	ABCG1	SR-B1	
MPM	2.5% HS^(d)^	10 – 26	71 – 78	71 – 78	13 – 23	0	0 – 9	[68]
AcLDL-loaded MPM	2.5% HS^(d)^	23 – 44	48 - 63	31 - 42	33 - 44	7 – 27	4 – 10	[68]
ABCA1^−/−(b)^: MPM	2.5% HS^(d)^	11	94	94	n.a.	0	6	[68]
ABCA1^−/−(b)^: AcLDL-loaded MPM	2.5% HS^(d)^	15	96	70	n.a.	26	4	[68]
ABCG1^−/−(b)^: MPM	2.5% HS^(d)^	18	70	70	23	n.a.	7	[68]
ABCG1^−/−(b)^: AcLDL-loaded MPM	2.5% HS^(d)^	29	46	46	50	n.a.	4	[68]
SR-B1^−/−(b)^: MPM	2.5% HS^(d)^	11	74	74	26	0	n.a.	[68]
SR-B1^−/−(b)^: AcLDL-loaded MPM	2.5% HS^(d)^	28	40	29	60	11	n.a.	[68]
mBMDM, AcLDL-loaded and GW3965-stimulated^(c),(r)^	2.5% MS^(e)^	6 – 8	n.d.	4 ^(p)^	40	15	0 ^(q)^	[69]
AcLDL-loaded MPM	2.5% HS ^(f)^	9	76	n.d.	18	n.d.	6	[70]
AcLDL-loaded J774	2.5% HS^(f)^	5	76	n.d.	14	n.d.	10	[70]
J774	2.8% apoB-depleted HS^(g)^	11	53	n.d.	17	n.d.	30	[71]
J774, stimulated by cAMP	2.8% apoB-depleted HS^(g)^	13	51	n.d.	39	n.d.	10	[71]
J774, stimulated by cAMP	2.8% apoB-depleted HS^(g),(h)^ (b.a. ^(i)^)	13	38	n.d.	60	n.d.	6	[71]
J774, stimulated by cAMP	2.8% apoB-depleted HS^(g),(h)^ (a.a. ^(i)^)	17	33	n.d.	65	n.d.	7	[71]
J774, stimulated by cAMP	2.8% apoB-depleted HS^(g),(j)^ (b.a. ^(k)^)	12	39	n.d.	53	n.d.	12	[71]
J774, stimulated by cAMP	2.8% apoB-depleted HS^(g),(j)^ (a.a. ^(k)^)	15	38	n.d.	57	n.d.	9	[71]
J774, stimulated by cAMP	2.8% apoB-depleted HS ^(g),(l)^ (b.a.^(m)^)	9	43	n.d.	52	n.d.	5	[71]
J774, stimulated by cAMP	2.8% apoB-depleted HS ^(g),(l)^ (a.a. ^(m)^)	13	36	n.d.	58	n.d.	2	[71]
J774, stimulated by cAMP	2.8% apoB-depleted HS^(g),(n)^ (b.a.^(o)^)	12	31	n.d.	56	n.d.	9	[71]
J774, stimulated by cAMP	2.8% apoB-depleted HS^(g),(n)^ (a.a. ^(o)^)	17	33	n.d.	62	n.d.	10	[71]

^(a)^ Efflux that was not inhibited by both probucol and BLT-1 (inhibitors of ABCA1 and SR-BI); it is assumed that uninhibited efflux is a sum of aqueous diffusion and ABCG1 pathways.

^(b)^ Cells were isolated from the knockout mouse.

^(c)^ The contributions of the pathways are roughly estimated by the comparison of the efflux in the cells from KO animal versus WT cells.

^(d)^ Serum was pooled from 15 normolipidemic individuals; cholesterol efflux for 8 hours.

^(e)^ Efflux for 2 hours.

^(f)^ Serum was pooled from 12 normolipidemic individuals; efflux for 4 hours.

^(g)^ 2.8% apolipoprotein B -depleted serum is equivalent to 2% serum. The serum was from healthy nonsmokers; efflux for 4 hours.

^(h)^ Serum is from female subjects with HDL-C = 45-51.

^(i)^ The data are grouped for 11 females with efflux below average (b.a.) and 11 females with efflux above average (a.a.).

^(j)^ Serum is from female subjects with HDL-C = 69-77.

^(k)^ The data are grouped for 9 females with efflux below average (b.a.) and 9 females with efflux above average (a.a.).

^(l)^ Serum is from male subjects with HDL-C = 36-40.

^(m)^ The data are grouped for 7 males with efflux below average (b.a.) and 7 males with efflux above average (a.a.).

^(n)^ Serum is from male subjects with HDL-C = 59-66.

^(o)^ The data are grouped for 8 males with efflux below average (b.a.) and 8 males with efflux above average (a.a.).

^(p)^ Calculated by subtraction values for other pathways from 100%.

^(q)^ Estimated using cell that were not treated with AcLDL and GW3965 since they have higher level of SR-B1 expression.

^(r)^ GW3965 is LXR agonist.

n.d.: not determined; n.a.: not applicable

AcLDL: acetylated LDL; HS: human serum; mBMDM: mouse bone marrow-derived macrophage; MPM: mouse peritoneal macrophages; MS: mouse serum.

## Abbreviations and Designations

ACAT: Acyl-coenzyme A:cholesterol acyltransferase
acLDL: Acetylated low-density lipoprotein
apoA-I: Apolipoprotein A-I
CE: Cholesteryl ester
ER: Endoplasmic reticulum
ERC: Endocytic recycling compartment
HCBS: High capacity binding site
HDL: High density lipoprotein
HEK: Human embryonic kidney
HMGCR-3: Hydroxy-3-methylglutaryl coenzyme A reductase
LD: Lipid droplet
LDL: Low-density lipoprotein
LDLR: LDL receptor
LE: Late endosome
L-FABP: Liver fatty acid-binding protein
LTP: Lipid transfer protein
LXR: Liver X receptor
MCSs: Membrane contact sites
OSBP: Oxysterol-binding protein
PI4P: Phosphatidylinositol-4-phosphate
PIP2: Phosphatidylinositol (4,5) bis-phosphate
PL: Phospholipid
PM: Plasma membrane
rHDL: Reconstituted high density lipoproteins
SCAP: SREBP cleavage-activating protein
SCP-2: Sterol carrier protein 2
SM: Sphingomyelin
SR-B1: Scavenger receptor class B type 1
SREBP: Sterol regulatory element-binding protein
STARD: StAR related lipid transfer domain containing proteins
TGN: Trans-Golgi network
VAP: Vesicle-associated membrane protein-associated protein
VLDL: Very density lipoprotein.
